# A retrospective study of pharmacological treatment in anorexia nervosa: 6-month and 12-month follow-up

**DOI:** 10.1186/s12888-023-04604-3

**Published:** 2023-02-27

**Authors:** Huei-Ping Chiu, Min-Wei Huang, Shr-Yu Tsai, Chiann-Yi Hsu

**Affiliations:** 1 Kaohsiung Municipal Kai-Syuan Psychiatric Hospital, Kaohsiung, Taiwan; 2 Department of Medical Education, Taichung Veterans General Hospital, Taichung, Taiwan; 3 Department of Psychiatry, Taichung Veterans General Hospital, Taichung, Taiwan; 4 Department of Physical Therapy and Graduate Institute of Rehabilitation Science, China Medical University, Taichung, Taiwan; 5 Department of Neurology, Taichung Veterans General Hospital, Taichung, Taiwan; 6 Biostatistics Task Force of Taichung Veterans General Hospital, Taichung, Taiwan

**Keywords:** Anorexia nervosa, Body mass index, Pharmacological treatment, Medication adherence

## Abstract

**Background:**

Anorexia nervosa (AN) is a serious and potentially life-threatening eating disorder characterized by starvation and malnutrition, a high prevalence of coexisting psychiatric conditions, marked treatment resistance, frequent medical complications, and a substantial risk of death. Body mass index (BMI) is a key measure of treatment outcome of AN and it is necessary to evaluate the long-term prognosis of AN. This study aimed to better assess the BMI course trend between different medications and timepoints in order to improve AN treatment in clinical practice.

**Methods:**

During the period 2010–2021, we retrospectively reviewed historical data of all patients diagnosed with AN. There were two groups in this study, which were based on the duration of follow-up. Group A was a 6-month follow-up group, comprising 93 patients (mean age 19.6 ± 6.8 years), with BMI assessed at three consecutive time points: first outpatient visit (T0), three months follow-up (T3), and six months follow-up (T6). Group B was a 12-month follow-up group comprising 36 patients (mean age 17.0 ± 5.2 years) with BMI assessed at five consecutive time points: first outpatient visit (T0), three months follow-up (T3), six months follow-up (T6), nine months follow-up (T9), and twelve months follow-up (T12). In our study, we retrospectively compared BMI courses based on patients’ usage of medication using the following variables: single medication, switching medications, combined medications, and without medications. The primary outcome measurement was BMI recorded at the 6-month follow-up and the 12-month follow-up respectively. In our study, which was conducted at Taichung Veterans General Hospital, we reviewed outpatient medical records of all patients with AN who were seen at the hospital during the period 2010–2021.

**Results:**

In Group A (6-month follow-up), patients treated with antidepressants showed a mean BMI increase of 1.3 (*p* < 0.001); patients treated with antipsychotics showed a mean BMI increase of 1.1 (*p* = 0.01); patients treated with switching medications showed a mean BMI increase of 0.1 (*p* = 0.397); patients treated with combined medications showed a mean BMI increase of 0.5 (*p* = 0.208); and patients treated without medications showed a mean BMI increase of 0.1 (*p* = 0.821). The results indicated that patients with AN had a significant BMI increase after treatment with antidepressants and antipsychotics in the 6-month follow-up group. In Group B (12-month follow-up), patients treated with antidepressants showed a mean BMI increase of 2.7 (*p *< 0.001); patients treated with antipsychotics showed a mean BMI increase of 2.8 (*p* = 0.168); patients treated with switching medications showed a mean BMI decrease of 0.8 (*p* = 0.595); patients treated with combined medications showed a mean BMI increase of 1.6 (*p* = 0.368); and patients treated without medications showed a mean BMI increase of 1.0 (*p* = 0.262). The results indicated that patients with AN had a significant BMI increase after treatment with antidepressants at the 12-month follow-up.

**Conclusions:**

AN is a complex disease caused by multiple factors. Evaluating its long-term prognosis is crucial. Our study provides insights and highlights three key findings: 1) medication adherence is crucial in treating AN, 2) frequent switching of medications may not promote weight gain and may also require a re-establishment of rapport with patients with AN, and 3) pharmacotherapy, especially antidepressants, is more effective than no treatment. Further research is needed to confirm these findings.

**Supplementary Information:**

The online version contains supplementary material available at 10.1186/s12888-023-04604-3.

## Introduction

Anorexia nervosa (AN) is a serious and potentially life-threatening eating disorder characterized by starvation and malnutrition, a high prevalence of coexisting psychiatric conditions, marked treatment resistance, frequent medical complications, and a substantial risk of death [1]. According to the Diagnostic and Statistical Manual of Mental Disorders, fifth edition (DSM-5) [2], AN is characterized by a restriction of energy intake, intense fear of gaining weight, and a disturbance in the way in which one’s body weight or shape is experienced. Furthermore, a number of individuals with AN bothered by body image distortion may fail to recognize the seriousness of their condition [3]. The two subtypes of AN are restricting type and binge-eating/purging type. The restricting subtype is associated with an earlier age of onset, a better prognosis, and a greater possibility of crossover to the other subtype [4, 5]. The binge-eating/purging subtype exhibits higher levels of core eating disorder (ED) psychopathology such as dietary restraint, eating concern, and shape/weight concerns [6]. The specific transition from restrictive-type anorexia nervosa (AN-R) to disorders involving binging and purging behaviors (BPB) is related to a worsening of the clinical picture and worse long-term outcomes [7]. There are several prognostic factors of long-term outcome in AN such as short duration of inpatient treatment, short duration of disorder, and preserved insight [8]. Zipfel et al. [9] suggested that longer duration of disorder before first inpatient treatment and lower body-mass index (BMI) were associated with a poor outcome, which indicates the importance of early identification and intervention.

BMI is a key measure of treatment outcome of AN [10]. It is imperative that the first-line approach in the management of AN be directed at weight gain and restoration of normal weight [11]. Based on the Anorexia Nervosa Treatment of Out-Patients (ANTOP) trial in Germany in 2014, a higher baseline BMI and shorter illness duration are strong positive predictors for a better outcome in outpatients with AN [12]. Current severity of AN is based on BMI according to the standard set by the World Health Organization (WHO Western Pacific Region 2000, as follows: mild, BMI greater than or equal to 17 kg/m2,moderate: BMI 16–16.99 kg/m2; severe: BMI 15–15.99 kg/m2, extreme: BMI less than 15 kg/m2. When treating patients with AN, the British guideline [13] NICE recommends helping patients to reach a healthy body weight or BMI, and states that weight gain is key in supporting other psychological, physical and quality of life changes that are needed for improvement or recovery. However, the effect of pharmacotherapy on body weight gain or BMI increase in patients with AN remains controversial. Most international guidelines recommend treatment for AN based on a multidisciplinary approach, including nutritional, somatic, psychiatric, and social components, and to use caution when prescribing medications to patients with AN, as they may lead to a number of common medical complications, such as heart problems, electrolyte imbalance, or bone loss [13–15]. In a recent multidisciplinary review of medication in AN [11], no psychotropic medication has proved efficacious in terms of weight gain, and there is only weak data showing it can alleviate certain negative psychological symptoms. Nonetheless, during the clinical course of AN treatment, relief of negative symptoms is important for the construct of therapeutic alliance (TA), which was found to be a reliable predictor of outcome for various disorders in some large meta-analyses, with a positive influence on outcomes [16, 17].

A number of major trials have been conducted to investigate the pharmacological treatment of AN. In light of the distinct psychological features in AN including the near-delusional quality of intense and irrational beliefs about body shape and weight [18], antipsychotics have been proposed as a potential therapeutic medication for AN. The second-generation antipsychotic (SGA) olanzapine is one of the most-studied medications in the treatment of AN because it is associated with substantial weight gain in other disorders, such as schizophrenia. Attia et al. [19] conducted a randomized, double-blind, placebo-controlled trial of 152 adult outpatients with AN and found a significant increase in BMI in the olanzapine group (0.259 versus 0.095 kg/m2 per month, respectively) compared to the placebo group. A recent meta-analysis and systematic review of a total of seven articles (304 patients with AN) revealed that olanzapine was effective in the treatment of AN with mean increased BMI 0.68 kg/m2 at the end of treatment in adults [20]. Antidepressants have also been considered for AN treatment due to symptoms of AN that overlap with other psychiatric disorders responsive to antidepressants, including major depressive disorder, obsessive–compulsive disorder, and anxiety disorders [21]. However, the role of antidepressants in the treatment of AN has largely been disappointing. In a case–control design study [22], no significant differences were found between the Mirtazapine group and controls with regard to weight (*P* = 0.981) or BMI (*P* = 0.576) in AN patients. Moreover, Holtkamp et al. [23] conducted a retrospective study of selective serotonin reuptake inhibitors (SSRIs) treatment in 32 adolescent females (mean age 14.5 ± 1.4 years) with AN, but the results showed insufficient evidence of efficacy in term of BMI and standardized BMI (*p* = 0.84), core eating disorder symptoms (Anorexia Nervosa Inventory for Self-Rating, *p* = 0.79), depression scores (Depressions-Inventar für Kinder und Jugendliche, *p* = 0.75), or obsessive–compulsive scores (Children's Yale-Brown Obsessive Compulsive Scale, *p* = 0.40). In a recent review article on the role of antidepressants in the treatment of adults with AN, the authors state that antidepressants should not be used as a single therapy for AN, although some SSRIs may prevent relapse and improve depressive and anxiety symptoms [24]. A small open-label study that compared sertraline with a placebo reported that sertraline improved depressive symptoms, perceptions of ineffectiveness, a lack of interoceptive awareness, and perfectionism, but not weight gain [25]. Overall, the effect of antidepressant in the treatment of AN still remains limited and inconsistent.

Since the rates of dropout from treatment for AN are high, ranging from 20.2% to 51% (inpatients) and from 29 to 73% (outpatients), patients with AN may consider switching medications or may discontinue a medication due to its side effects or apparent effects on certain personality dimensions such as impulse control, self-efficacy, maturity fear, among others [26, 27]. Although numerous studies have been conducted on the pharmacological treatment of AN, few studies have compared differences in BMI trends among patients receiving a single medication, combined medications or switching medications during the clinical course of AN. In the current study, we retrospectively reviewed the data of patients diagnosed with AN and compared the BMI course based on medication usage, i.e., a single medication, switching medications, combined medications, and without medications. The primary outcome measurement was BMI recorded at the 6-month follow-up and the 12-month follow-up respectively. This study aimed to better understand the BMI course trends based on the different patterns of medication usage at various time points in order to improve AN treatment in clinical practice.

## Materials and methods

### Patient population

During the period 2010–2021, we retrospectively reviewed the historical data of all patients diagnosed with AN according to the Diagnostic and Statistical Manual of Mental Disorders, 4th Edition (DSM-IV), DSM-5, the International Classification of Diseases, 10th Revision (ICD-10), or the International Classification of Diseases, 11th Revision (ICD-11). All data were collected from outpatient records at Taichung Veterans General Hospital, and the standard of care for patients with AN at the hospital is based on evidence-based guidelines and clinical experience. We only included data from outpatient records and did not include any records from inpatient treatment in our study. There were no patients in our study who received inpatient treatment before transitioning to outpatient treatment.

There were two groups in this study based on the duration of the follow-up period. Group A was a 6-month follow-up group, which comprised 93 patients (mean age 19.6 ± 6.8 years) whose BMI was assessed at three consecutive time points: first outpatient visit (T0), three months’ follow-up (T3), and six months’ follow-up (T6). Group B was a 12-month follow-up group comprising 36 patients (mean age 17.0 ± 5.2 years) whose BMI was assessed at five consecutive time points: first outpatient visit (T0), three months’ follow-up (T3), six months’ follow-up (T6), nine months’ follow-up (T9), and twelve months’ follow-up (T12). All descriptive data are listed in Table 1. Additionally, we adjusted the baseline BMI (AN severity) by using repeated measures ANOVA in both groups. Please refer to Table 2 for the results.

**Table 1:** Descriptive characteristics of two groups

	**Group A 6-month follow-up**		**Group B 12-month follow-up**	
	*n* = 93	(%)	*n* = 36	(%)
**Age** (mean ± SD)	19.63	± 6.80	17.00	± 5.20
**Gender**				
female	86	(92.47%)	33	(91.67%)
male	7	(7.53%)	3	(8.33%)
**BMI** (mean ± SD)				
T0	14.59	± 2.44	14.74	± 2.22
T3	14.89	± 2.43	14.78	± 2.38
T6	15.32	± 2.43	15.65	± 2.35
T9	-	-	16.12	± 2.54
T12	-	-	16.51	± 2.69
**Group**				
antidepressant	42	(45.16%)	17	(47.22%)
antipsychotics	5	(5.38%)	2	(5.56%)
combined meds	11	(11.83%)	2	(5.56%)
switching meds	5	(5.38%)	4	(11.11%)
without meds	30	(32.26%)	11	(30.56%)

**Table 2:** Adjusted Baseline BMI (T0) of two groups

Group A: 6-month follow-up (adjusted BMI T0)										
	n		T3				T6			*p* value
			Mean		±SD		Mean		±SD	
Group										0.021*
antidepressant	42		14.9		±2.6		15.6		±2.6	
antipsychotics	5		14.1		±2.6		15.2		±2.7	
switching meds	5		14.3		±2.2		14.8		±1.6	
combined meds	11		14.7		±2.6		14.8		±2.7	
without meds	30		15.3		±2.2		15.3		±2.3	
Group B: 12-month follow-up (adjusted BMI T0)										
		T3		T6		T9		T12		
	n	Mean	±SD	Mean	±SD	Mean	±SD	Mean	±SD	*p* value
Group										0.006**
antidepressant	17	15.0	±2.7	16.4	±2.6	17.0	±2.5	17.5	±2.8	
antipsychotics	2	13.9	±3.5	15.8	±3.2	16.0	±3.5	16.9	±2.3	
switching meds	4	13.7	±2.0	14.5	±1.7	13.9	±2.1	13.5	±1.6	
combined meds	2	12.6	±2.2	12.9	±2.8	13.9	±3.3	14.7	±3.3	
without meds	11	15.4	±1.8	15.4	±1.8	16.0	±2.1	16.3	±2.0	

Repeated measure ANOVA

This research was approved by the ethics committee of Taichung Veterans General Hospital and conducted in accordance with Good Clinical Practice procedures and the current revision of the Helsinki Declaration.

#### With drug treatment

Patients with drug treatments were allocated into four categories as follows: with antidepressants, with antipsychotics, switching medication, and combined medication. In Group A (6-month follow-up group), 63 patients were treated with medications, with 42 of these patients with antidepressants, 5 patients with antipsychotics, 5 patients with switching medication, and 11 patients with combined medication. In Group B (12-month follow-up group), 25 patients were treated with medications, with 17 of these patients with antidepressants, 2 patients with antipsychotics, 4 patients with switching medication, and 2 patients with combined medication. The choice of antidepressants included four SSRI, i.e., fluoxetine, paroxetine, escitalopram, and sertraline, and one noradrenergic and specific serotonergic antidepressant (NaSSA), i.e., mirtazapine. There was a single choice of antipsychotic medication: sulpiride.

#### Without drug treatment

There were patients diagnosed with AN who did not receive psychotropic treatment. In Group A (6-month follow-up group), 30 out of 93 patients (32.26%) did not receive psychotropic treatment. In Group B (12-month follow-up group), 11 out of 36 patients (30.56%) did not receive psychotropic treatment.

### Assessment of BMI

According to the DSM-5, the diagnostic criteria for AN included restriction of energy intake relative to requirements, leading to a significantly low body weight. Level of severity of AN was based on BMI according to the standard set by the WHO Western Pacific Region 2000, follows: mild, BMI greater than or equal to 17 kg/m^2^; moderate, BMI 16–16.99 kg/m^2^; severe, BMI 15–15.99 kg/m^2^; extreme, BMI less than 15 kg/m^2^. We obtained a series of BMI data from outpatients' medical records.

### Statistical analysis

Repeated measures ANOVA was conducted to analyze the BMI measurements taken at the start of medication (T0), at the 3-month follow-up (T3), at the 6-month follow-up (T6), at the 9-month follow-up (T9), and at the 12-month follow-up (T12). The Bonferroni test was used for post-hoc analysis, and IBM SPSS version 22.0 was used for statistical calculations.

To ensure the statistical validity of the numbers for Group A (6-month follow-up) and Group B (12-month follow-up) in this study, we used the G*Power software with the following parameters: t-tests as the test family, linear bivariate regression as the statistical test (two groups with different intercepts), compromise power analysis (calculating implied alpha and power), one-tailed test, 93 subjects in Group A and 36 subjects in Group B, and default values for the remaining parameters. The software calculation indicated that the power (1-beta error probability) is 0.8637804.

## Results

### BMI measurements

**Fig. 1:**
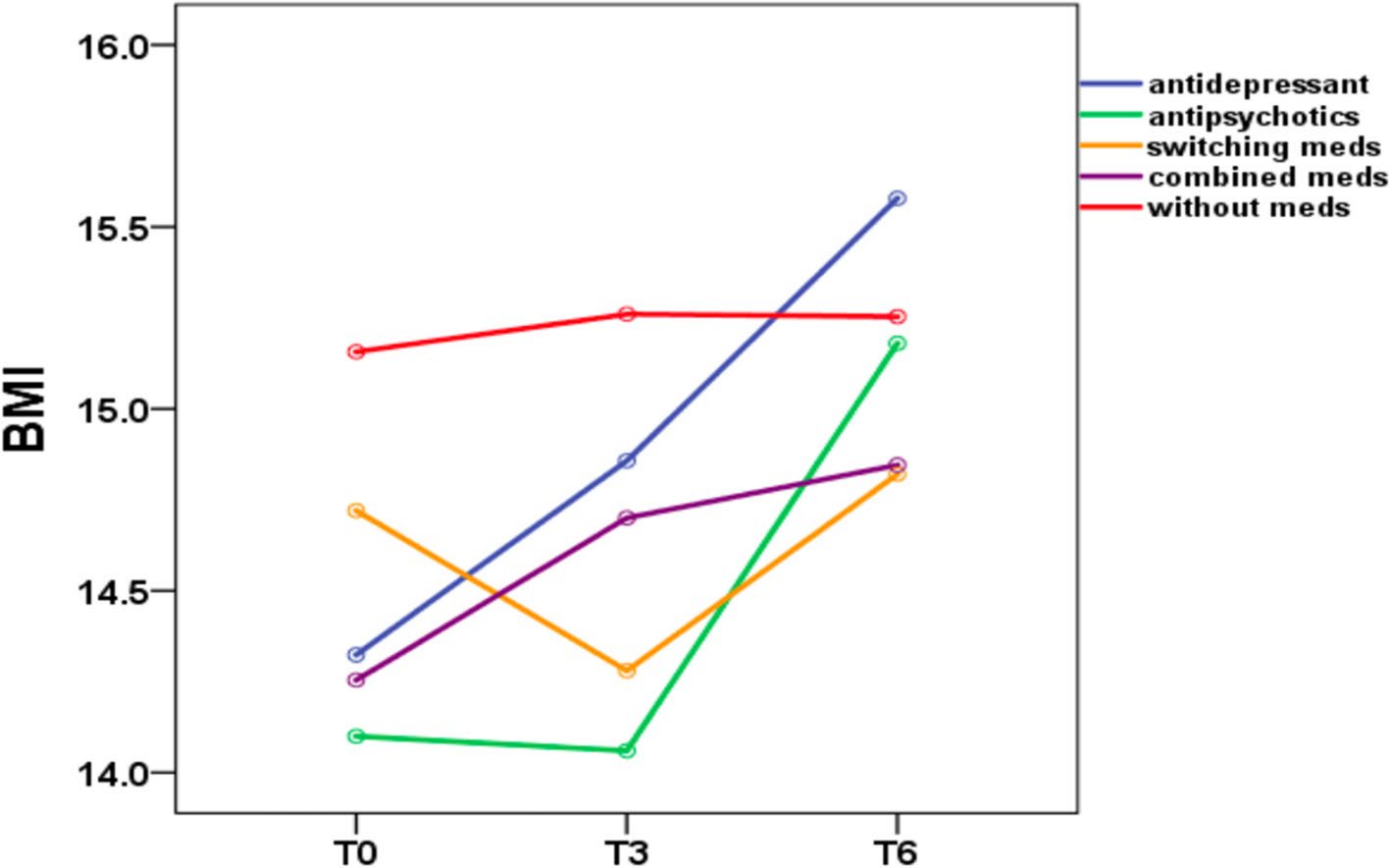
Body mass index group comparison of patients with anorexia nervosa during the 6-month outpatient follow-up

**Fig. 2:**
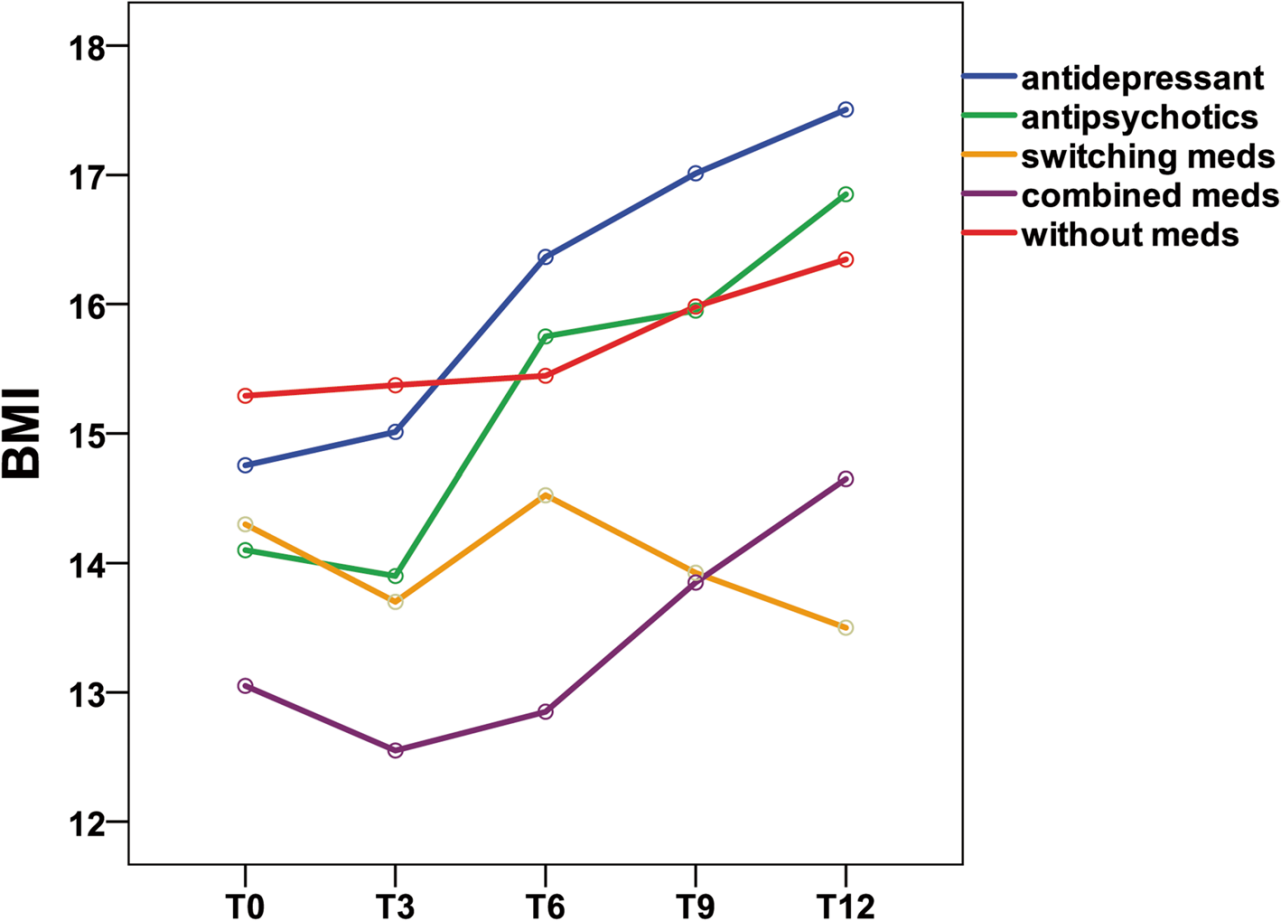
Body mass index group comparison of patients with anorexia nervosa during the 12-month outpatient follow –up

Table 3 shows the mean and standard deviation of BMI in Group A at three time points (T0, T3, and T6). Table 4 shows the mean and standard deviation of BMI in Group B at five time points (T0, T3, T6, T9, and T12). Figures 1 and 2 show the trends in BMI over time in line graphs for Group A and Group B, respectively.

**Table 3:** BMI differences in groups during the 6-month follow-up

BMI											
	n	T0		T3		T6		*p* value	Bonferroni		
		Mean	± SD	Mean	± SD	Mean	± SD		T0 vs T3	T0 vs T6	T3 vs T6
Total	93	14.6	± 2.4	14.9	± 2.4	15.3	± 2.4	< 0.001**	0.028*	< 0.001**	< 0.001**
Group											
antidepressant	42	14.3	± 2.8	14.9	± 2.6	15.6	± 2.6	< 0.001**	0.015*	< 0.001**	< 0.001**
antipsychotics	5	14.1	± 2.3	14.1	± 2.6	15.2	± 2.7	0.010*	1.000	0.072	0.090
switching meds	5	14.7	± 1.7	14.3	± 2.2	14.8	± 1.6	0.397	0.533	1.000	1.000
combined meds	11	14.3	± 2.2	14.7	± 2.6	14.8	± 2.7	0.208	0.814	0.477	1.000
without meds	30	15.2	± 2.2	15.3	± 2.2	15.3	± 2.3	0.821	1.000	1.000	1.000

*BMI* body mass index, *T0* first outpatient visit, *T3* three months follow-up, *T6* six months follow-up

**Table 4:** BMI differences in groups during the 12-month follow-up

		n		T0			T3			T6			T9			T12				*p* value	
				Mean	± SD		Mean		± SD	Mean		± SD	Mean		± SD	Mean		± SD			
Total		36		14.7	± 2.2		14.8		± 2.4	15.7		± 2.3	16.1		± 2.5	16.5		± 2.7		< 0.001**	
Group																					
antidepressant		17		14.8	± 2.6		15.0		± 2.7	16.4		± 2.6	17.0		± 2.5	17.5		± 2.8		< 0.001**	
antipsychotics		2		14.1	± 3.0		13.9		± 3.5	15.8		± 3.2	16.0		± 3.5	16.9		± 2.3		0.168	
switching meds		4		14.3	± 1.6		13.7		± 2.0	14.5		± 1.7	13.9		± 2.1	13.5		± 1.6		0.595	
combined meds		2		13.1	± 1.5		12.6		± 2.2	12.9		± 2.8	13.9		± 3.3	14.7		± 3.3		0.368	
without meds		11		15.3	± 1.8		15.4		± 1.8	15.4		± 1.8	16.0		± 2.1	16.3		± 2.0		0.262	
Bonferroni																					
T0 vs T3	T0 vs T6		T0 vs T9			T0 vs T12		T3 vs T6			T3 vs T9			T3 vs T12			T6 vs T9		T6 vs T12		T9 vs T12
1.000	0.016*		0.007**			0.001**		0.002**			0.002**			< 0.001**			0.100		0.004**		0.081
1.000	0.004**		0.001**			< 0.001**		< 0.001**			0.002**			< 0.001**			0.157		0.008**		0.122
1.000	0.577		1.000			1.000		0.855			0.155			1.000			1.000		1.000		1.000
1.000	1.000		1.000			1.000		1.000			1.000			1.000			1.000		1.000		1.000
1.000	1.000		1.000			1.000		1.000			1.000			1.000			1.000		1.000		–-
1.000	1.000		1.000			1.000		1.000			1.000			1.000			1.000		0.302		1.000

*BMI* body mass index, *T0* first outpatient visit, *T3* three months follow-up, *T6* six months follow-up, *T9* nine months follow-up, *T12* twelve months follow-up

#### Six-month follow-up

During the 6-month outpatient follow-up (Table 3), patients treated with antidepressants showed a mean BMI increase of 1.3 (*p* < 0.001); patients treated with antipsychotics showed a mean BMI increase of 1.1 (*p* = 0.01); The BMI increase was statistically significant (*p* ≤ 0.05) in the antidepressant and antipsychotic groups. In contrast, patients treated with switching medications showed a mean BMI increase of 0.1 (*p* = 0.397); patients treated with combined medications showed a mean BMI increase of 0.5 (*p* = 0.208); and patients treated without medications showed a mean BMI increase of 0.1 (*p* = 0.821). The BMI increase was not statistically significant (*p* ≥ 0.05) in the medication switching, medication combination and without medication groups. In the Bonferroni post hoc test, patients treated with antidepressants showed a significant BMI difference between the following time periods T0 vs. T3, T0 vs. T6, and T3 vs. T6. No significant BMI difference among the other four groups (with antipsychotics, switching medications, combined medications, without medications) emerged, while the antidepressant group showed a significant difference in BMI for the time periods T0 vs. T3 (*p* = 0.015), T0 vs. T6 (*p* < 0.001), and T3 vs. T6 (*p* < 0.001).

#### Twelve-month follow-up

During the 12-month outpatient follow-up (Table 4), patients treated with antidepressants showed a mean BMI increase of 2.7 (*p* < 0.001).The BMI increase was statistically significant (*p* ≤ 0.05) in the antidepressant group.

However, patients treated with antipsychotics showed a mean BMI increase of 2.8 (*p* = 0.168); patients treated with switching medications showed a mean BMI decrease of 0.8 (*p* = 0.595); patients treated with combined medications showed a mean BMI increase of 1.6 (*p* = 0.368); and patients treated without medications showed a mean BMI increase of 1.0 (*p* = 0.262). Obviously, the BMI increase was not statistically significant (*p* ≥ 0.05) in the antipsychotic, medication switching, medication combination and without medication groups. In the Bonferroni post hoc test, patients treated with antidepressants showed a significant BMI difference between the following time period: T0 vs. T6, or T0 vs. T9, T0 vs. T12, T3 vs. T6, T3 vs. T9, T3 vs. T12, and T6 vs. T12. No significant BMI difference among the other four groups (with antipsychotics, switching medications, combined medications, without medications) emerged, while the antidepressant group showed a significant difference for the following time periods: T0 vs. T6 (*p* = 0.004), or T0 vs. T9 (*p* = 0.001), T0 vs. T12 (*p* < 0.001), T3 vs. T6 (*p* < 0.001), T3 vs. T9 (*p* = 0.002), T3 vs. T12 (*p* < 0.001), and T6 vs. T12 (*p* = 0.008).

### Adjusted baseline BMI (T0)

In our study, the sample size for switching medication, combined medication, and without medication was small (5/11/30 in Group A and 4/2/11 in Group B) which impacted the statistical power of comparing the groups. To address this, we grouped the samples into two categories: staying on antidepressant or antipsychotic and switching, combining, or not taking medication. Using repeated measures ANOVA and adjusting for BMI at baseline (T0), we found that sticking with antidepressant or antipsychotic medication resulted in a statistically significant increase in BMI at 6 months in Group A (*p *= 0.022) and 12 months in Group B (*p* = 0.004). This suggests that staying on antidepressant or antipsychotic is more effective in increasing BMI compared to switching, combining, or not taking medication. Please refer to Table 5 for more details.

**Table 5:** Adjusted baseline BMI in two groups

**Group A: 6-month follow-up BMI (adjusted BMI T0)**										
	n		T3				T6			*p* value
			Mean		±SD		Mean		±SD	
Group										0.022*
antidepressant and antipsychotic	47		14.8		±2.6		15.5		±2.6	
switching, combined and without medication	46		15.0		±2.2		15.1		±2.3	
**Group B: 12-month follow-up BMI (adjusted BMI T0)**										
	n	T3		T6		T9		T12		*p* value
		Mean	±SD	Mean	±SD	Mean	±SD	Mean	±SD	
Group										0.004**
antidepressant and antipsychotic	19	14.9	±2.7	16.3	±2.5	16.9	±2.5	17.4	±2.7	
switching, combined and without medication	17	14.6	±2.1	14.9	±1.9	15.2	±2.3	15.5	±2.3	

Repeated measure ANOVA

## Discussion

To our knowledge, this is the first study to retrospectively review BMI courses at five timepoints (at the beginning of treatment and at 3, 6, 9, and 12 months after treatment) in outpatients with AN receiving different medications. In our study, patients who adhered to their antidepressant or antipsychotic medication regimens had a significant BMI increase in the 6-month follow-up, compared with patients who switched medication, used combined medication or did not use medication. These findings suggest that medication adherence to a single medication may play a key role in improving BMI in both the antidepressant and antipsychotic groups. Our study highlights the importance of medication adherence, and the essential role of pharmacotherapy in the treatment of AN. The contributions of this study are further elaborated in the following sections.

First, based on the results of our study, it seems that medication adherence is more important than the specific medication in the treatment of patients with AN. Since the core symptoms of AN are in direct conflict with the medical goal of weight gain, adherence to the therapeutic recommendations presents significant clinical challenges [28]. In the 6-month follow-up, we found that patients with AN had significant BMI increase after treatment with antidepressants (*p* < 0.001) and antipsychotics (*p* = 0.01). However, no significant differences in BMI were found in patients who switched medication, used combined medication or did not use medication. The results suggest that maintaining a consistent medication regimen may be more effective at increasing BMI, compared to switching medications or using a combination of medications. On the other hand, psychoeducational interventions to enhance medication adherence among patients with AN is critical during the treatment course. Since the main treatment of AN as delineated in the current international guidelines is a form of psycho-behavioral therapy which can be provided on an outpatient basis [13–15], specific psychological therapies such as trans-diagnostic Cognitive Behaviour Therapy – Enhanced (CBT-E) are the first-line treatment for all eating disorders and have the greatest impact on symptom reduction and other outcomes [29]. Novick et al. [30] found that insight, therapeutic alliance, and adherence are closely related and all of these factors have an impact on clinical and functional status in patients. That being said, pharmacotherapy may only play an adjunctive role in the treatment of AN, and behavior change and medication adherence are the keys to recovery. Patients with AN have been particularly impaired by poor insight [31], as this disorder is characterized by distorted cognitions of weight and body shape as well as ambivalence in motivation to recover [32]. Level of insight has been demonstrated to be of clinical relevance in the treatment and prognosis of psychiatric disorders [33]. Based on our results, we speculate that medication adherence is mainly accompanied by better quality of insight to the disorder itself, and increased insight may lead to acceptance of weight gain in the clinical course of AN while receiving medication.

Additionally, our study found that patients treated with antidepressants had a significant increase in BMI in the first 3 months (T0-T3) and the second 3 months (T3-T6). This information may be useful for clinicians in evaluating the effectiveness of medication based on weight change in patients with AN after prescription. A study reviewed outpatient therapy for patients with AN and found that patients with the greatest early weight gain had significantly higher levels of remission [34]. Thus, close monitoring in weight change may help clinicians to adjust the treatment plan accordingly, and it must be kept in mind that the association between early weight gain trajectories and the outcome of the disorder seems to be crucial. However, our findings are inconsistent with the results of recent studies on use of antidepressants in the treatment of AN. In general, there is a lack of solid evidence that antidepressants can improve weight gain in the treatment of patients with AN [35]. In our study, the choice of antidepressants included four SSRIs, namely fluoxetine, paroxetine, escitalopram, and sertraline, and one NaSSA mirtazapine. Fluoxetine is one of the most-studied SSRIs in AN and seems to have the most evidence supporting its use in the treatment of AN in weight-restored individuals [24]. An open trial [36] investigated fluoxetine use in 6 patients with chronic refractory AN-R previously treated with tricyclic antidepressants (TCAs), trazodone, and/or monoamine oxidase inhibitors (MAOIs), and found that fluoxetine treatment (mean duration = 7.6 months) was not only associated with significant improvement of depressive symptoms in all patients, but was also associated with significant weight gain in 5 patients (83.3%) and improvement in obsessive–compulsive symptoms. Kaye et al. [37] conducted an open trial of patients with AN who were followed up for 11 ± 6 months and found a positive effect of fluoxetine on BMI development when administered after at least partial weight recovery. However, in the study no control subjects were investigated, and the results were comparable with data from the literature. In contrast, Holtkamp et al. [23] challenged the efficacy of SSRI medication in the treatment of adolescent AN. In the study, both SSRI and non-SSRI groups showed a similar course of BMI at the 3-month and 6-month follow-up. The inconsistent evidence on the effectiveness of antidepressants in treating AN patients necessitates the need for additional studies with a larger sample size and longer follow-up duration.

Antipsychotics have also been suggested as a potential treatment option for AN. In our study, the use of antipsychotics was found to result in a significant increase in BMI after 6 months, but not after 12 months. This may be due to the limited sample size in the 12-month follow-up, the choice of antipsychotics, and the fact that antipsychotics did not address the comorbid depression and anxiety associated with AN. It is worth noting that the small sample size in the antipsychotic group (*n* = 2) compared to the antidepressant group (*n* = 17) may have limited the power of this result. Additionally, the antipsychotic used in our study was sulpiride, rather than the more commonly studied second-generation antipsychotic (SGA) olanzapine. Few studies on first-generation antipsychotics (FGAs) have been conducted on AN patients due to its severe side effects, such as grand mal seizure, which may occur in patients taking chlorpromazine [38]. A double-blind, placebo-controlled, cross-over study [39], which included 18 female AN inpatients revealed that sulpiride was superior to placebo for daily weight gain, especially in the first treatment period of three weeks. However, in the cross over analysis, this effect did not reach statistical significance. The absence of supporting evidence for the effectiveness of sulpiride in treating AN patients necessitates the need for more robust and high-quality research in order to offer practical guidance to clinicians.

Second, our study found that the BMI of patients in the switching medication group did not increase. The results suggest that frequent switching of medications may not be beneficial for weight gain and may also require a re-establishment of rapport with patients with AN. Switching medications can have a significant impact on the patient-doctor relationship, as it often involves discussing the current treatment plan, evaluating its effectiveness, and making changes to better meet the patient's needs. This process requires open communication, trust, and collaboration between the patient and doctor, which can help to re-establish and strengthen the relationship between them. However, there is a possibility that the poorer outcome in the "switch medication" group may simply reflect that this group was more medication resistant, rather than the switch itself causing the poorer outcome. Unless the clinical situation requires a medication change, prescribers may take steps to optimize current medication regimens (e.g., dosage adjustments, behavioral or psychosocial interventions) before switching medications [40]. In clinical practice, taking the time to understand a patient's motivations for wanting to discontinue or switch medications and approaching medication changes with caution can be beneficial. This is because changes in medication often result in the need to re-establish the patient-doctor relationship.

Third, in our study, pharmacotherapy was found to be superior to no medication in treating AN patients..—Compared to the group without medication, the antidepressant group showed a statistically significant increase in BMI at both the 6-month and 12-month follow-up, while the antipsychotics group showed a significant increase in BMI at the 6-month follow-up. This may be due to the fact that antidepressants effectively address the underlying depression and anxiety issues in AN patients. Although recent studies on pharmacotherapy show inconsistent evidence regarding improvements in weight gain in patients with AN, a number of the symptoms frequently associated with AN, such as depression and anxiety are responsive to medications [21]. As recommended by most guidelines, it is important to consider the overall picture of the patient, including their psychiatric, medical, nutritional, and social circumstances. Medication should be prescribed on the basis of the clinical evaluation and this evaluation should always include the patient’s opinion about the treatment [11]. Apart from specific psychological therapy, the treatment needs to be provided by a multidisciplinary team to address important nutritional, physical and mental health comorbidities [41].

It is important to note that, due to the naturalistic study design (different medications at different dosages, non-randomized), our findings are preliminary and should be interpreted with caution. There are also several limitations to this study that should be considered. First, although we assessed BMI, our study lacked other clinical evaluations commonly seen in AN, such as eating disorder psychopathology, depressive symptomatology, and obsessive–compulsive symptomatology. Besides, since patients with AN are prone to have other psychiatric and medical comorbidities, the complete information of these comorbidities may be further addressed in detail but it is lacking in our study. Second, our study lacked long-term BMI follow-up. The observation periods of the BMI course were short, with follow-up at 6 months and 12 months only. Third, our study encompassed a relatively limited number of subjects (*n* = 93 in the 6-month follow-up group and *n* = 36 in the 12-month follow-up group). Therefore, further well-controlled studies with a larger sample size and a longer follow-up period are required to confirm our findings. Fourth, there are many other possible factors that might be related to BMI fluctuation in patients with AN. For example, the poorer outcome in the "switch medication" group may simply reflect that this group was more medication resistant, rather than the switch itself causing the poorer outcome. Whether patients receive nonpharmacological interventions (psychotherapy, family therapy, etc.) or whether patients receive medical treatments from internal medicine specialists may contribute to BMI change. Comprehensive information of all kinds of treatment for patients with AN should be considered in the future study. Fifth, in our study, we considered patients with AN who came back to the outpatient department routinely for prescription are those patients who were taking medication regularly. However, patients with AN are notorious for their poor medication compliance, so appropriate measurement of medication adherence such as self-report questionnaires or structured interviews should be included.

## Conclusions

AN is a disease caused by various factors. It is necessary to evaluate the long-term prognosis of AN. This study provides a direction that warrants further exploration. Our study highlights three key findings: 1) medication adherence is more critical than the specific medication in treating AN patients, 2) frequent switching of medications may not promote weight gain and may also require a re-establishment of rapport with patients with AN, and 3) pharmacotherapy, particularly the use of antidepressants, is more beneficial than no medication at all in addressing the depression and anxiety symptoms in AN patients. Further studies with a larger sample size and longer follow-up period are required to confirm our findings.

## Supplementary Information

**Additional file 1. **Group A: patients with AN during 6-month outpatient follow-up (*n*=93). Group B: patients with AN during 12-month outpatient follow-up (*n*=36)

## Data Availability

All data generated or analyzed during this study are included in this published article and its supplementary information files.

## Abbreviations

AN: Anorexia nervosa
BMI: Body mass index
DSM-5: Diagnostic and Statistical Manual of Mental Disorders, fifth edition
ED: Eating disorder
AN-R: Restrictive-type anorexia nervosa
BPB: Binging and purging behaviors
ANTOP: Anorexia Nervosa Treatment of Out-Patients
WHO: World Health Organization
TA: Therapeutic alliance
SSRIs: Selective serotonin reuptake inhibitors
DSM-IV: Diagnostic and Statistical Manual of Mental Disorders, 4th Edition
ICD-10: The International Classification of Diseases, 10th Revision
ICD-11: The International Classification of Diseases, 11th Revision
NaSSA: Noradrenergic and specific serotonergic antidepressant
CBT-E: Cognitive Behaviour Therapy – Enhanced
TCAs: Tricyclic antidepressants
MAOIs: Monoamine oxidase inhibitors
SGA: Second-generation antipsychotic
FGAs: First-generation antipsychotics
